# Pet Humanisation and Related Grief: Development and Validation of a Structured Questionnaire Instrument to Evaluate Grief in People Who Have Lost a Companion Dog

**DOI:** 10.3390/ani9110933

**Published:** 2019-11-07

**Authors:** Stefania Uccheddu, Loriana De Cataldo, Mariangela Albertini, Stanley Coren, Goncalo Da Graça Pereira, Anouck Haverbeke, Daniel Simon Mills, Ludovica Pierantoni, Stefanie Riemer, Lucia Ronconi, Ines Testoni, Federica Pirrone

**Affiliations:** 1 Vet Ethology, 3090 Overijse, Belgium; uccheddus@gmail.com (S.U.); anouck.haverbeke@vetethology.be (A.H.); 2 Department of Philosophy, Sociology, Education and Applied Psychology, University of Padua, 35122 Padua, Italy; decataldolory@virgilio.it (L.D.C.); l.ronconi@unipd.it (L.R.); ines.testoni@unipd.it (I.T.); 3 Department of Veterinary Medicine, University of Milan (UNIMI), 20133 Milan, Italy; federica.pirrone@unimi.it; 4 Department of Psychology, University of British Columbia, Vancouver, V5V 3K3, Canada; scoren@psych.ubc.ca; 5 Center for Animal Knowledge, 1495-020 Lisbon, Portugal; ggp.vet@gmail.com; 6 Escola Superior Agrária de Elvas (ESAE), Instituto Politécnico de Portalegre (IPP), 7350-092 Elvas, Portugal; 7 Odisee University College, Salto Research Group, Hospitaalstraat 23, 9100 Sint-Niklaas, Belgium; 8 School of Life Sciences, University of Lincoln, Lincoln, Lincolnshire LN6 7DL, UK; dmills@lincoln.ac.uk; 9 Veterinary Behaviour & Consulting Services at CAN Training Centre, 80128 Naples, Italy; ludovica.pierantoni@gmail.com; 10 Companion Animal Behaviour Group, Division of Animal Welfare DCR-VPHI Vetsuisse Faculty, University of Bern, 3012 Bern, Switzerland; stefanie.riemer@vetsuisse.unibe.ch

**Keywords:** grief, dog, dog-human bond

## Abstract

**Simple Summary:**

The aim of this study was the scientific validation of a novel instrument (the Mourning Dog Questionnaire) designed to allow a comprehensive quantitative analysis of grief responses in dog owners after the death of a pet dog, which is a still underestimated issue. This instrument was based on a grid of quantitative measurable outcomes that combines five different questionnaires concerning levels of attachment to pets, responses to the loss of a pet, outlook on life events and grief, perceptions of animals in terms of emotions, needs and legal right. We found that pet owners have the tendency to humanise their pet and perceive animals no differently from humans in terms of emotions, needs and legal rights. In addition, after the death of the pet, a negative view of life was described according to the findings. The Mourning Dog Questionnaire could be a useful tool considering the high numbers of people who are at risk of experiencing the loss of a companion dog, which makes this type of grief as potential major concern for public health and human welfare.

**Abstract:**

People often develop strong emotional connections with their dogs and consider them to be members of the family. The purpose of this study was to develop a novel validated tool, the Mourning Dog Questionnaire, to recognise and evaluate the mourning process in people who have lost a dog. The research model was based on a grid of five different questionnaires: the Pet Bereavement Questionnaire, the Lexington Attachment to Pets Scale, the Animal-Human Continuity Scale, the Positivity Scale, and the Testoni Death Representation Scale. The Italian version of the survey was posted on social networks. A sample of 369 Italian dog owners filled in the questionnaire (mean age ± SD 42.00 ± 10.70 years). Reliability indices were good for all instruments. The total scores of the five questionnaires correlated with each other. The results from the Mourning Dog Questionnaire support the negative view of life after the death of a pet and people’s tendency to humanise their pet, since dog owners perceived animals no differently from humans in terms of emotions, needs and legal rights. Findings arising from the use of the Mourning Dog Questionnaire will help the implementation of rationality-based strategies to improve the wellbeing, resilience and quality of life of people in the world experiencing the loss of a pet.

## 1. Introduction

In humans, bereavement is a natural and common event caused by the loss of a significantly loved person through death [1]. According to the standard definition, the term grief refers to the emotions that accompany bereavement, whereas mourning refers to the behaviour that social groups expect following bereavement when a family member has died [1]. People often develop strong emotional connections with their dogs to the point that nearly all dog owners in the Western World consider them to be members of the family [2,3]. Owners are attached to their pet dogs mostly because the relationship provides the same, or an even greater, sense of comfort and security, and reliable affection as close human relationships [3]. According to Cowles [4], the degree of attachment between owner and pet determines the psychological impact on the owner resulting from the death of a pet [5]. Not surprisingly, when a pet dies, some owners can experience significant grief over this loss [4]. Gerwolls and Labott [5] found that levels of grief following a pet’s loss were comparable to levels of grief following the loss of a human, while according to Hunt et al. [6] the period of grief associated with the loss of a companion animal may be even longer than with a person [7]. In the study by Rajaram et al. [6], people who had recently lost a pet were three times more likely to report symptoms of depression than the normal population. The acute phase of grief reactions after the death of a familiar pet often occurs within a two-month time frame around the loss, with uncomplicated pet grief lasting from six months to a year [8]. Subclinical symptoms of sadness and grief may also be present and persist for six months or even longer [8]. However, there are a number of variables that notably influence the experience [7] of human adults during dog loss, including the nature and quality of the human-pet attachment bond, the quality of social support available to the bereaved, and the circumstances of the pet’s death [8,9]. In addition, gender and age of the owner [9], cause of loss [10] and animal species [10] have been indicated as potential influential factors of grief intensity and predictors of extreme grief. Life events, which can be classified either as positive or negative stressors, can also play a role in the way a person handles loss [11]. 

The death of a pet can produce effects similar to those caused by a variety of other losses, including a spouse, a child, health or a job [7,12]. Indeed, more severe grief was reported by bereaved pet owners who had experienced more stressful life events than by those who had experienced fewer stressful life events [10,13]. It should also be noted that dogs live much shorter lives than humans. As a result, dog owners may experience multiple losses leading to repeated grief, which might overwhelm the person’s normal coping ability [7]. In addition, due to the short lifespans of most companion animals, the loss of a pet may be a child’s first experience of death, which has been shown to help them explore their own connections towards not only pets, but also their relatives and friends [14].

Despite the relatively high frequency and intensity of grief over the death of a pet among owners, this process is largely unrecognised socially and underexplored in research [8]. To date, only a few studies have focused specifically on grief-related loss of pet dogs, and they are limited either by the modest samples [5], the conceptualisation of an individual’s grief solely within an attachment framework [12,15], or the lack of a standardised, well-validated measure designed specifically to assess pet loss-related bereavement [16,17,18,19].

For this reason, the purpose of the present study was to develop and validate a novel instrument (the Mourning Dog Questionnaire-MDQ) to identify and measure people’s grief over the loss of a companion dog. In particular, we wanted to test the hypothesis that dog-human attachment and the humanisation of pet dogs was not only associated with a perspective associated with continuity between humans and non-human animals (concerning emotions, needs, legal rights) but also with a pessimistic vision of life following the death of the pet.

Assuming that a relationship exists between a person’s humanisation of their pet, the psychological impact of losing a pet and pet attachment, the MDQ provides a research model based on a grid of quantitative measurable outcomes combining different previously validated questionnaires (Pet Bereavement Questionnaire [6], Lexington Attachment to Pets Scale [20], Animal-Human Continuity Scale [21], Positivity scale [22], Testoni Death Representation Scale [23]), which reflect and translate the actual state (i.e., physical, emotional) of an individual into objective data that can be agreed upon by different observers. 

In this study we present the results obtained from the statistical validation process of the Italian version of the MDQ, followed by an analysis of correlations among all the constructs and between the constructs and participant/pet characteristics. 

## 2. Materials and Methods 

### 2.1. Study Design

The instrument (presented in Appendix A) is composed of two parts. The first part is divided into two sections: (1) owner demographics, including gender, age, educational level, marital status, household composition and presence of children, occupational status; (2) information on the deceased dog, including age, sex, time since dog’s death, length of ownership, cause of death, and whether or not the owner lived alone at the time of the death.

The second part is composed of a combination of five questionnaires, namely the Pet Bereavement Questionnaire (PBQ) [6], the Lexington Attachment to Pets Scale (LAPS) [20], the Animal-Human Continuity Scale (AHCS) [21], the Positivity Scale (P-Scale) [22] and the Testoni Death Representation Scale (TDRS) [24], which, individually, have already been demonstrated to have good internal reliability. The Italian versions of PBQ, LAPS and TDRS have already demonstrated good factor structure and good construct validity (the degree to which all questionnaires are measuring appropriately) [24]. All instruments, except for TDRS, have been forward- and backward-translated by two independent translators [25]. 

Briefly, the PBQ is a 16-item 4-point Likert-type scale which assesses pet bereavement distress. The PBQ is composed of three distinct factors: Grief (items: 2, 3, 5, 7, 10, 12, 15), Anger (items: 1, 4, 11, 13, 14), and Guilt (items 6, 8, 9, 16). The LAPS is a 23-item scale measuring pet attachment. Respondents answer questions on a 0–3 Likert-type scale for each of the following factors: General Attachment (items: 10, 11, 12, 13, 15, 17, 18, 19, 21, 22, 23), Animals Substituting People (items: 1, 2, 4, 5, 6, 7, 9), and Animal Rights/Animal Welfare. The last factor assesses a pet’s perceived status within the household (items: 8, 14, 16, 20). The AHCS is a 12-item 7-point Likert-type animal attitude scale related to philosophical/religious/world view about whether there is a qualitative difference between humans and other animals. It measures the extent to which the respondents view humans and animals as on the same continuum or in a dichotomous fashion. Items 1, 2, 3, 7, 9, 10, 11 and 12 are worded in a dichotomous direction and scoring is reversed; items 4, 5, 6, 8 are worded in the continuous direction. The scale is composed of three factors: Rational Capacity (item 1, 2, 3, 9, 12), Superiority versus Equality (item 7, 9, 10), and Evolutionary Continuum (item 4, 5, 6). The P-Scale is an 8-item unidimensional 5-point Likert type scale designed to measure positivity, that is, the tendency to view life and experiences with a positive outlook. Lastly, the TDRS is a 6-item self-report measure which assesses the attitudes of individuals toward the ontological representation of death as a passage to an afterlife or as a form of annihilation. Lower scores indicate that the individual represents death as a passage, whereas people with higher scores represent death as total annihilation.

### 2.2. Participants

The survey was published on the internet and social networks (e.g., Facebook), targeting Italian participants who were older than 21 years and had experienced the death of a dog. Moreover, to meet key requirements for enrolment of a larger project of which this study is part, all the recruited dog owners had at least two dogs at the time of the dog’s death. The study was approved by the University of Padova Ethics Committee (46DF1164A03D63129CEE38D7571F8FB7). Participants were given written information about the aim and the procedures of the study and the right to withdraw at any time. In addition, they were assured that the survey was anonymous, and confidentiality would be maintained by the researchers. Before data collection, written informed consent was obtained from each participant. Participation was voluntary. 

### 2.3. Statistical Analysis

After creating initial descriptive summaries, we explored item response distributions of the questionnaires (PBQ, LAPS, AHCS) that had not been previously validated in Italian and we performed a Confirmatory Factor Analysis (CFA), hypothesising that items should group into the same construct as in the original version of each questionnaire. Given the ordinal nature of the data, we used the Diagonally Weighted Least Squares (DWLS) robust estimator. Several fit indices were considered to evaluate models: Comparative Fit Index (CFI), Tucker-Lewis Index (TLI), Root-Mean-Square Error of Approximation (RMSEA), and Weighted Root Mean Square Residual (WRMR). Cut-off values for adequate fit for CFI and TLI were > 0.90, RMSEA < 0.06 [26], and WRMR < 1.00 [13]. The reliability of all the construct scales was also evaluated by Cronbach’s alpha (estimate of reliability). Finally, we examined the correlations between all the construct scales and between each scale and the characteristics of the participants and the characteristics of the companion animals using Pearson and point-biserial correlation. Normality, asymmetry and kurtosis of the data were evaluated based on plots and statistical indexes, respectively, which showed only minor deviations, confirming the appropriateness of parametric analyses.

Analyses were carried out using SPSS 24 (IBM Corp. Released 2016. IBM SPSS Statistics for Windows, Version 24.0. Armonk, NY.) for descriptive, reliability and correlations analysis and using the R package lavaan [27] for CFAs.

## 3. Results

We collected reports from 369 Italian dog owners (329 females and 38 males) with a mean age of 42.00 ± 10.70 (SD) years (range 21.00–70.00). In total, 17.60% reported their highest level of qualification to be post-graduate, 32.80% a university degree, 45.30% a high-school qualification, and 4.30% a middle school award. Regarding marital status at the time of loss, 61.80% participants were married or in a long-term commitment relationship, 24.40% were single and alone, 7.30% were separated/divorced and alone, 19.80% lived with at least one child (age 0–14) but no other adult. 

When the dog died, 18.20% of respondents were living alone. About 67.00% of participants reported having lost their dog over one year ago, 21.40% less than 6 months ago, and 11.70% between 6 and 12 months before participating in the survey. The average length of the deceased dog’s ownership was 9.70 ± 4.50 (SD) years (range 1.00–18.00); the dog’s mean age at death was 11.40 ± 3.90 (SD) years (range 1.00–19.00). With respect to the circumstances of the dog’s death, 48.80% of the respondents declared that it was unexpected, while 58.00% had opted for euthanasia.

Most of the items showed a left-skewed distribution (skewness ranging between −1.73 to −0.45 for 6 items of PBQ; between −7.06 to −0.42 for 19 items of LAPS; between −1.16 to −0.82 for 2 items of AHCS), however some items showed a right-skewed distribution (skewness ranging between 0.52 to 2.47 for 7 items of PBQ; between 2.55 to 4.45 for 2 items of LAPS; between 1.19 to 5.96 for 7 items of AHCS), [28].

The confirmatory factor analysis results indicate a good fit of the hypothesised factor structure for each questionnaire (Table 1). Factor loadings were significant at the 0.001 level in all the CFA models except for one item in the LAPS model (item 21) and four items in the AHCS model (items: 4, 5, 8, 12). Standardised coefficients ranging between 0.31 and 0.90 with an average factor loading of 0.61 for PBQ; ranging between 0.23 and 0.78 with an average factor loading of 0.55 for LAPS; ranging between 0.28 and 0.73 with an average factor loading of 0.46 for AHCS.

Reliability indices were good for all the instruments, with Cronbach’s alpha coefficients ranging between 0.51 and 0.88 (PBQ Grief alpha = 0.85; PBQ Anger alpha = 0.63; PBQ Guilt alpha = 0.71; PBQ Total alpha = 0.83; LAPS Animal Rights alpha = 0.69; LAPS People Substituting alpha = 0.76; LAPS General Attachment alpha = 0.74; LAPS Total alpha = 0.85; AHCS Total alpha = 0.51; P-Scale alpha = 0.84; TDRS alpha = 0.88).

Table 2 shows the correlations between the instruments. The PBQ Total score positively correlated with the LAPS Total score; furthermore, the PBQ Grief and Anger factors positively correlated with all the LAPS factors and the LAPS Total score, while the PBQ Guilt factor only positively correlated with the LAPS People Substituting and with the Total score. 

The AHCS positively correlated with all the LAPS factor and its Total score, and positively correlated with PBQ Grief and Anger factors and the PBQ Total score, but it did not correlate with the PBQ Guilt factor.

The P-Scale negatively correlated with the LAPS Total score and with all the LAPS factors except for the General Attachment factor. It negatively correlated with the PBQ Grief and Guilt factors and positively correlated with PBQ Anger factor and the PBQ Total score. The P-Scale negatively correlated with the AHCS.

The TDRS negatively correlated with the LAPS Total score and all its factors except for General Attachment. It was negatively correlated with the PBQ Grief factor and positively correlated with the P-Scale. Table 3 shows the correlations between constructs and participant/pet characteristics. With respect to participants’ characteristics, age was positively correlated with LAPS general attachment and AHCS Total score and negatively correlated with the all the LAPS scores. Education was negatively correlated with all the LAPS scores, PBQ Grief, PBQ Anger, PBQ Total and AHCS. Education was only positively correlated with P-scale Total. Occupation was negatively correlated with all the LAPS scores except LAPS Animal rights/welfare.

Among the dog-related characteristics, time since dog’s death correlated negatively with LAPS General attachment, LAPS Total and PBQ Grief, but correlated positively with TDRS Total. Time owning the dog correlated only negatively with PBQ Anger. Unexpected dog’s death positively correlated with all PBQ scores except PBQ Guilty. Living alone when a dog died negatively correlated only with PBQ Anger. Euthanasia correlated negatively with all the PBQ scores except PBQ Grief.

## 4. Discussion

In the present study, we aimed to develop and validate a multidimensional tool which can be used to obtain reliable quantitative assessment of owners’ grief over the loss of a companion dog, taking into account the tendency to humanise the pet, the psychological impact of losing a pet and pet attachment. The MDQ was shown to be a reliable and valid instrument for use with dog owners who have lost a pet dog.

As for all surveys which include only people who use the Internet, this study might have sampling biases compared to the general population. Familiarity with Internet technology is not uniform across demographic, cultural, and geographic groups [29]. Internet users tend to be richer and better educated than non-Web users [30]. Accordingly, the relatively high education level of respondents is not surprising. There was also a clear gender bias (96% female), but this too was expected [31,32]. Women have been reported to be more empathic and to have a higher concern for animal welfare compared with men [32,33]. Thus, females may also be more willing to fill out online surveys on sensitive issues relating to pets than males. 

In relation to the CFA, it has been suggested that RMSEA values less than 0.05 are good, between 0.05 and 0.08 acceptable, between 0.08 and 0.10 marginal, and values greater than 0.1 poor [34]. The PBQ constructs provided an acceptable fit to the data (RMSEA = 0.056), whereas LAPS and AHCS did produce a good fit (RMSEA = 0.034, RMSEA = 0.038 respectively). The CFI value was close to 0.9 for PBQ, LAPS, AHCS, indicating a relatively good fit [35] using this criterion. The other fit indices, TLI, should be over 0.90 for a good fit [35] and, in this sample, the three instruments meet this criterion. WRMR values of 1.264 (PBQ), 1.086 (LAPS), 1.026 (AHCS) are just above the values of <1.00 suggested as indicative of adequate model fit [36]. Overall, based on all the indices, we suggest that the current sample has an acceptable fit. Cronbach’s alpha values of between 0.51 and 0.88 on subscales and total scores indicate that the Italian version of the questionnaires were reliable in their structure. 

All the total scores of the constructs were related to each other (PBQ, LAPS, AHCS, P-Scale, TDRS). Testoni reported a high correlation between PBQ and TDRS [32] as evidence that all these variables, which are typically described in Complicated grief in relation to human loss, also characterise pet loss. 

Great variation existed among individuals in how they reacted to companion animal death, and the reason of this variation still remains to be well understood. People vary considerably in how they can manifest their grief [9], ranging from sorrow to a suicidal reaction [37]. Research into people’s response to the death of a companion animal is scarce and mostly focused on identifying factors, such as gender, age, education and marital status, which may help predict who is likely to experience extreme or pathological grief (e.g., [10,19,38,39,40]). Women are reported to show more intense grief responses than males [10,16,40,41,42] and also more likely to seek support services [43]. This is consistent with females’ general tendency to report stronger negative (but also positive) emotions than males [44]. Given the sample bias in our sample, the current data should not be considered norm reference values; nonetheless, the internal relationships identified can be considered valid. In our study, women’s answers correlated positively with Grief items in PBQ and total PBQ, but not with other items, which is consistent with previously cited findings of a more evident grief reaction after the death of a pet in women. Nevertheless, it is unclear whether bereaved female owners experience more extreme grief than their male counterparts or whether they are simply more ready and willing to report it. 

Although age has shown significant associations with grief in previous studies [9], the findings are not entirely consistent. For example, McCutcheon [10] reported that younger adults were more susceptible to intense grief than older owners, whereas Quackenbush and Glickman [42] found that young adults suffered less grief than either adolescents or adults over the age of 40. Other researchers have found that age was not predictive of the extent of grief [16,41]. In our instrument, younger age correlated positively with LAPS general attachment and LAPS animal rights and welfare but negatively with PBQ Anger and PBQ Guilty items. Although we only considered bivariate associations because our sample size and number of independent variables would potentially make a multivariate analysis unreliable, we can hypothesise about the nature of the relationships evident here, with a view to specific hypothesis testing in future studies. A strong bond not characterised by the feeling of guilt seems to characterise young humans. Hunt [6] interpreted the negative correlation between age and guiltiness from the perspective of loss inevitability. In this respect, young individuals have less experience of the loss of pets and may create strong attachment bonds with several dogs [45] which may be characterised by deep social synchrony [46]. The lack of experience may make them feel more guilty as they may be more idealistic and feel they should be able to do more. On the other hand, older individuals have a reduced tendency to feel guilty [6], possibly because they spend more time taking care of their pets compared to other family members [6] and also because they have more experience of what might be realistic. Our findings show negative correlations (Table 1) between education level and three LAPS factors (general attachment, people substituting and animal rights/welfare). A negative correlation was also found between PBQ grief and PBQ Anger, but not among these two constructs and PBQ Guilt items. In a previous study, Templer [21] reported negative correlations between education and AHCS Total and P-scale Total score. In line with the previous literature, our findings support the interpretation that highly educated people express lower levels of attachment, grief and anger. We suggest that this might be associated with knowledge-based expectations. However, no correlation was found with Guilt, which may be based more on the objective circumstances of loss. In this regard, it is important to note how pet owners’ social environment might affect their response to companion animal death. Housing conditions and the presence of children did not correlate with any elements of our questionnaires, which is in contrast to previous reports in other non-Italian cultures [8,15,41,43]. Owners who lived alone were normally reported to be more susceptible to extreme grief [10,41], as are those who report having little social support available [39]. Having no children has also been considered to increase risk [16,42]. Other factors, including the length of time a person has owned the deceased dog [38,42] and whether the person owned other pets when that dog died, were sometimes found to be associated with the extent of a grief response, although not consistently [16,19]. In our study, the length of time that the person had owned the dog was found to be negatively correlated with PBQ’s Anger items. This might be explained by a long relationship being able to reduce the feeling of anger after a death as has been described for conjugal bereavement [47]. When considering the modality of death, our results confirmed what has been previously reported in the literature, indicating convergent validity which means agreement among previous and recent findings: an unexpected death of the pet was related to intense feelings of anger and guilt, whereas pet euthanasia was associated with higher attachment and grief [24]. In the literature, there is evidence that the cause of a pet death (e.g., accidental death or euthanasia versus natural death) affects the likelihood of intense bereavement [10,40], in that owners who have decided to have their pet euthanised by a veterinarian experience significantly less grief compared to owners whose pet dies naturally. This can be explained by the suggestion that, in the latter case, many owners may not have time to prepare themselves for the pet’s death or to receive adequate support (for example from the veterinarian) [10].

## 5. Conclusions

People may be at risk for extreme grief responses upon the death of their companion animal [47]. In fact, the main factors related to grief for human beings (being guilty, in grief, in anger, with intrusive thoughts) are often present after the loss of a pet [31]. Correlations among PBQ, LAPS and TDRS support results previously obtained by Testoni [22] concerning the humanisation of pets. From this perspective, our correlations between PBQ, LAPS and TDRS with AHCS may not be surprising, as they show that dog owners tend to view animals and humans as being on the same continuum rather than as separated entities. As expected, P-scale scores (which measure the tendency to view life and experiences with a positive outlook) were negatively correlated with negative dimensions of grief: guilt, grief, anger, intrusive thoughts and decisional regrets that are captured from the respective questionnaires (PBQ and TDRS). Thus the MDQ can be considered a helpful and reliable tool to recognize and evaluate grief in people who have lost a companion dog through death. This may enable the implementation of rationality-based strategies to improve wellbeing and resilience in the family system, especially in the early stages that follow death, when the disabling consequences may be greatest. 

## Figures and Tables

**Table 1:** Fit indices of the Confirmatory Factor Analysis for each questionnaire.

Questionnaire	*N*	Factors	Chi^2^	Df	*p*-Value	CFI	TLI	RMSEA [90% CI]	WRMR
PBQ	369	3	217.247	101	<0.001	0.962	0.955	0.056 [0.046–0.066]	1.264
LAPS	369	3	325.446	227	<0.001	0.971	0.967	0.034 [0.026–0.042]	1.086
AHCS	369	1	82.175	54	0.008	0.934	0.920	0.038 [0.020–0.053]	1.026

Notes: PBQ = Pet Bereavement Questionnaire. LAPS = Lexington Attachment to Pets Scale. AHCS = Animal Human Continuity Scale. CFI = Comparative Fit Index; TLI = Tucker-Lewis Index; RMSEA = Root Mean Square of Approximation; WRMR = Weighted Root Mean Square Residual.

**Table 2:** Descriptive statistics and correlations between all constructs (*N* = 369).

Constructs		Range	M	SD	1	2	3	4	5	6	7	8	9	10	11
Lexington Attachment to Pets Scale (LAPS)															
1.	General attachment	0–33	28.91	3.65											
2.	People substituting	0–21	13.49	4.63	0.56 ***										
3.	Animal rights/welfare	0–15	13.69	1.85	0.52 ***	0.49 ***									
4.	Total score	0–69	56.09	8.52	0.85 ***	0.89 ***	0.71 ***								
Pet Bereavement Questionnaire (PBQ)															
5.	Grief	0–21	14.84	4.66	0.43 ***	0.39 ***	0.28 ***	0.46 ***							
6.	Anger	0–15	3.57	3.14	0.18 **	0.27 ***	0.16 **	0.26 ***	0.46 ***						
7.	Guilt	0–12	4.35	3.43	−0.01	0.20 ***	0.06	0.12 *	0.17 **	0.43 ***					
8.	Total score	0–48	22,76	8,46	0.30 ***	0.40 ***	0.24 ***	0.40 ***	0.79 ***	0.80 ***	0.66 ***				
Animal Human Continuity Scale (AHCS)															
9.	Total score	12–84	67.70	7.72	0.276 **	0.20 ***	0.48 ***	0.33 ***	0.29 ***	0.15 **	0.09	0.25 ***			
Positive Scale (P-Scale)															
10.	Total score	8–40	28.74	5.73	−0.04	−0.17 **	−0.13 *	−0.14 **	−0.17 **	−0.25 ***	−0.15 **	−0.24 ***	−0.11 *		
Testoni Death Representation Scale (TDRS)															
11.	Total score	6–30	19.71	6.32	−0.08	−0.18 **	−0.11 *	−0.16 **	−0.11 *	0.05	−0.03	−0.005	−0.09	−0.14 **	
Constructs					1	2	3	4	5	6	7	8	9	10	11

Note. Reliability Cronbach’s alpha coefficients are in diagonal. * *p* < 0.05; ** *p* < 0.01; *** *p* < 0.001.

**Table 3:** Correlations between constructs and participant/dog characteristics.

Characteristics	LAPS General Attachment	LAPS People Substituting	LAPS Animal Rights/Welfare	LAPS Total	PBQ Grief	PBQ Anger	PBQ Guilt	PBQ Total	AHCS Total	P-Scale Total	TDRS Total
***Participant’s characteristics***											
Gender	0.08	0.06	0.02	0.07	0.14**	0.08	−0.01	0.10 *	0.06	−0.01	−0.06
Age	0.13 *	−0.03	0.18 **	0.08	−0.08	−0.16 **	−0.17 **	−0.17 **	0.11 *	0.06	−0.04
Education	−0.17 **	−0.15 **	−0.18 **	−0.19 ***	−0.20 ***	−0.12*	0.02	−0.15 **	−0.19 ***	0.13 *	0.04
Occupation	−0.11 *	−0.13 *	−0.08	−0.14*	0.00	0.05	0.01	0.02	0.01	−0.01	−0.02
Housing condition	−0.06	−0.01	0.05	−0.02	−0.06	−0.04	−0.04	−0.06	0.05	0.02	0.04
Presence of children	−0.03	−0.04	−0.09	−0.06	−0.02	−0.05	0.03	−0.02	−0.02	0.01	0.06
*Dog’s characteristics*											
Time from dog’s death	−0.11 *	−0.09	−0.09	−0.11 *	−0.17 **	−0.01	0.00	−0.10	−0.05	0.09	0.14 **
Dog’s keeping time	0.04	−0.02	0.00	0.01	−0.07	−0.15 **	−0.01	−0.09	0.01	0.06	0.00
Unexpected dog’s death	0.06	0.03	0.04	0.05	0.13 *	0.11 *	0.03	0.13*	0.04	0.07	−0.04
Lived alone when dog died	−0.08	−0.03	0.04	−0.04	−0.05	−0.11 *	−0.06	−0.09	0.02	0.03	0.06
Dog’s euthanasia	0.04	−0.03	−0.05	−0.01	−0.06	−0.27 ***	−0.14 **	−0.19 ***	−0.04	−0.04	−0.08

Note. For Gender 0 = male, 1 = female; Age (years); Education 0 = Low, 1 = High; Occupation 0 = No worker, 1 = Worker; Housing condition 0 = With others, 1 = Alone; Presence of children 0 = No, 1 = Yes; Time from dog’s death 0 = Up to 12 months, 1 = Over 12 months; Dog possession time (years); Unexpected dog’s death 0 = No, 1 = Yes; Dog’s euthanasia 0 = No, 1 = Yes; * *p* < 0.05; ** *p* < 0.01; *** *p* < 0.001.

## Appendix A

**Owner Demographics**
Gender: female/male/prefer not to say
Age (in years)
City
Country
State (for USA citizens)
Highest level of education achieved: primary school/ high school- secondary education/Tertiary level education, e.g., Degree or other form of graduate level education (e.g., BSc, BA, HND)/Post-graduate education (e.g., PhD, MSc, MA)
Marital status: single/married- long term committed relationship/divorced—separated/other
Household composition: one person/multi person
Your role in the household: parent- primary caregiver/daughter—son/single person family/housemate/other
Presence of children in the household: yes/no
Number of children (0–14 years old) in the home: 0/1/2/3/4/more than 4
Age of the youngest child (days/weeks/months/years, please specify)
Age of the oldest child (months/years, please specify)
House with yard/garden: yes/no
What do you do for a living?
**The deceased dog**
Gender: female/male
Sexual status: intact/entire- neutered/desexed
Age at acquisition: before 50 days/ 50–70 days/ 71–90 days/91 days–1 year/more than 1 year/homebred
Age at death (months/years, please specify)
Size: small (less than 10 kg)/medium (10–25 kg)/ large (25–40 kg)/giant (more than 40 kg
Source: shelter/breeder/stray/pet shop/private (friend, relative)/other
Main reason you acquired/adopted this dog: companionship/security/precursor of children/for the children/the dog needed a home/Special needs (i.e., seeing eye dog, therapy animal)/work needs/dog sports/other
When did your animal pass away?: less than 6 months ago/6–12 months ago/more than 12 months ago
Was the death expected?: yes/no
Were you living alone when your dog died?: yes/no
Which of the following best describes the role filled by this animal in your relationship?: partner/friend/source of protection/working animal/family (baby)/family (brother/sister)/simply an animal
Was the dog euthanased? Yes/no
Reason for euthanasia: dog’s health issue/dog’s behavioural issue/owner’s issue/the dog was not euthanased
In case of euthanasia for behavioural issues, can you please specify the cause:
How long has the dog lived with you (months/years, please specify)?
Was this your first dog?: yes/no
In your opinion, to this dog you were: simply a human/ a friend/ a group member/other—positive relationship/other—negative relationship
**Lexington Attachment to Pets Scale** Please consider the following statements to the pet that has passed away. Before answering, take a few minutes to think about your dog, your relationship and the life you have shared together. Read the following statements referring to this relationship, although the statements are formulated in the present tense, consider the relationship you had with your dog and indicate if you strongly agree, agree, neither agree or disagree, disagree, or strongly disagree with each statement by circling the appropriate answer. 1 = strongly disagree 2 = disagree 3 = neither agree or disagree 4 = agree 5 = strongly agree
My pet means more to me than any of my friends
Quite often I confide in my pet
I believe that pets should have the same rights and privileges as family members
I believe my pet is my best friend
Quite often, my feelings toward people are affected by the way they react to my pet
I love my pet because he/she is more loyal to me than most of the people in my life
I enjoy showing other people pictures of my pet
I think my pet is just a pet
I love my pet because he/she never judges me
My pet knows when I am feeling bad
I often talk to other people about my pet
My pet understands me
I believe that loving my pet helps me stay healthy
Pets deserve as much respect as humans do
My pet and I have a very close relationship
I would do almost anything to take care of my pet
I play with my pet quite often
I consider my pet to be a great companion
My pet makes me feel happy
I feel that my pet is a part of my family
I am not very attached to my pet
Owning a pet adds to my happiness
**Animal-Human Continuity Scale** Directions: Please answer each of the following questions as honestly as you can. Use the scale provided below. Choose only one answer. 1 = Strongly Disagree 2 = Moderately Disagree 3 = Slightly Disagree 4 = Unsure Slightly 5 = Agree 6 = Moderately Agree 7 = Strongly Agree
I consider my pet to be a friend
Humans have a soul but animals do not.
Humans can think but animals cannot.
People have a life after death but animals do not.
People are animals.
Animals are afraid of death.
People evolved from lower animals.
People are superior to animals.
Animals can fall in love.
People have a spiritual nature but animals do not.
The needs of people should always come before the needs of animals.
It’s okay to use animals to carry out tasks for humans.
It’s crazy to think of an animal as a member of your family.
**The Positivity Scale** The following statements describe how a person might act in certain situation. 1 = strongly disagree 2 = disagree 3 = neither agree or disagree 4 = agree 5 = strongly agree
I have great faith in the future
I am satisfied with my life
Others are generally here for me when I need them
I look forward to the future with hope and enthusiasm
On the whole, I am satisfied with myself
At times, the future seems unclear to me
I feel I have many things to be proud of
I generally feel confident in myself
**Testoni Death Representation Scale** The following questionnaire is the result of various interviews held with many participants and it deals with the most common forms of representing death and the relative thoughts. There are no “exact answers”: this questionnaire considers only personal opinions. Please: think for a moment what death and dying means to you and answer the following questions.
EVALUATION 1 = absolutely disagree 2 = disagree 3 = indifferent 4 = agree 5 = absolutely agree
Death is only a passage. After my death, I will continue to be and I will continue to remember all experiences of this life.
Death is a definitive annihilation. After my death I will cease to exist, therefore I won’t have any experiences.
Death is a radical change. After my death I will no longer be conscious of being myself.
Death is a definitive annihilation. After my death, even if others remember me, I won’t remember anything.
Death is only a passage. After my death, I will continue to exist and therefore I will have experiences.
Death is a radical change. After my death I will have experiences that will not have any connection with my present life.
